# BCL2L15 Depletion Inhibits Endometrial Receptivity via the STAT1 Signaling Pathway

**DOI:** 10.3390/genes11070816

**Published:** 2020-07-17

**Authors:** Diqi Yang, Ai Liu, Yanqin Wu, Bin Li, Sha Nan, Ruiling Yin, Hongmei Zhu, Jianguo Chen, Yi Ding, Mingxing Ding

**Affiliations:** College of Veterinary Medicine, Huazhong Agricultural University, Wuhan 430070, China; diqiyang@mail.hzau.edu.cn (D.Y.); liuai@webmail.hzau.edu.cn (A.L.); wyq1003@webmail.hzau.edu (Y.W.); binli.bl1996@gmail.com (B.L.); nansha@webmail.hzau.edu.cn (S.N.); yinrl@webmail.hzau.edu.cn (R.Y.); zhuhongmei@mail.hzau.edu.cn (H.Z.); chenjg@mail.hzau.edu.cn (J.C.); dingyi@mail.hzau.edu.cn (Y.D.)

**Keywords:** BCL2L15, STAT1, hormone, endometrial receptivity, goats

## Abstract

In domestic ruminants, endometrial receptivity is critical for a successful pregnancy and economic efficiency. Although the endometrium undergoes major cellular changes during peri-implantation, the precise mechanisms regulating goat endometrial receptivity remain unknown. In this study, we investigated the functional roles and signal transduction of the B-cell lymphoma 2 (Bcl-2)-like protein 15 (BCL2L15) in the regulation of endometrial receptivity in vitro. Our results showed that BCL2L15 was up-regulated in goat endometrial epithelial cells (EECs) under progesterone (P_4_), estradiol (E_2_), and interferon-tau (IFN-τ) treatments. Our knockdown of BCL2L15 by specific shRNA that significantly hampered endometrial receptivity. In the absence of BCL2L15, the signal transducer and activator of transcription (STAT)1 and STAT3 pathway were activated. Additionally, pretreatment with the STAT1 inhibitor, fludarabine, restored the effect of silencing BCL2L15 on the endometrial receptivity, but not the STAT3 inhibitor Stattic. Overall, these results suggested that BCL2L15 is the key regulator of endometrial receptivity in goats, regulating the endometrial receptivity through the STAT1 pathway. Understanding the function of BCL2L15-STAT1 in endometrial receptivity is important to the exploration of new targets for the diagnosis and treatment of early pregnancy failure, and improving the success rates for artificial reproduction.

## 1. Introduction

In ruminants, early pregnancy failure is a significant impediment to improving the reproductive rates and animal husbandry development [1]. Early pregnancy failure occurs when a free-floating mature embryo attaches to the endometrium when it is in an aberrant receptive state. Considering the importance of endometrial receptivity during embryo implantation, the regulatory mechanism of endometrial receptivity must be revealed. Several molecules and signaling pathways activated in endometrial epithelial cells (EECs) help establish a receptive endometrium. Members of the signal transducer and activator of transcription (STAT) family can induce multiple signal responses to participate in physiological and pathological processes [2,3]. The STAT family contains seven members, including STAT1 to STAT4, STAT5a, STAT5b, and STAT6 [4]. During the “window of implantation” (WOI), the STAT pathways are significantly involved in the successful maintenance of pregnancy via regulation of the epithelial polarity, epithelial–mesenchymal interactions, stromal decidualization, and cell proliferation [5,6]. In previous studies, researchers demonstrated that interferon tau (IFN-τ)-mediated the endometrial receptivity via STAT1 [7,8]. In pigs, STAT1 was induced in EECs via estrogen [9]. Keigo’s research found that the mRNA expression of STAT1 was up-regulated in bovine EECs by day 15 and 17 of pregnant exosome treatment [10].

B-cell lymphoma 2 (Bcl-2)-like protein 15 (BCL2L15), also known as BCL2 family kin (BFK), belongs to the BCL2 protein family, and appears to induce weak apoptosis and antagonize the pro-survival function of Bcl2 [11,12]. Previous studies reported BCL2L15 expression in the stomach, ovaries, bone marrow, spleen, and uterus [13]. Researchers found that the expression of BCL2L15 was reduced during malignant transformation in colon cancer samples [14]. The knockdown of BCL2L15 promoted tumor growth, and cell proliferation was reported in colorectal cancer research [15]. BCL2L15 was identified as an exosomal protein from ewe uterine flushings on day 17 of pregnancy [10]. In addition, Zhang et al., using transcriptome analyses, reported that the highest level of BCL2L15 was observed in the receptive endometrium (RE), rather than the pre-receptive endometrium (PE) by strand-specific Ribo-Zero RNA-Seq and quantitative-PCR in goats [16].

Although BCL2L15 has been found to participate in many key roles in regulating physiological or pathological processes, there are no studies showing that that BCL2L15 is directly involved in regulating the endometrial receptivity in goats. We therefore hypothesized that BCL2L15 may be required to produce the receptive endometrium. To examine this hypothesis, we assessed the effectiveness of BCL2L15 on cell proliferation, cell adhesion, and microvilli in EECs. Our results suggested that BCL2L15 is the key regulator of endometrial receptivity, through the STAT1 pathway. 

## 2. Results

### 2.1. Hormone and *IFN-τ* Treatment Triggered BCL2L15

**Figure 1:**
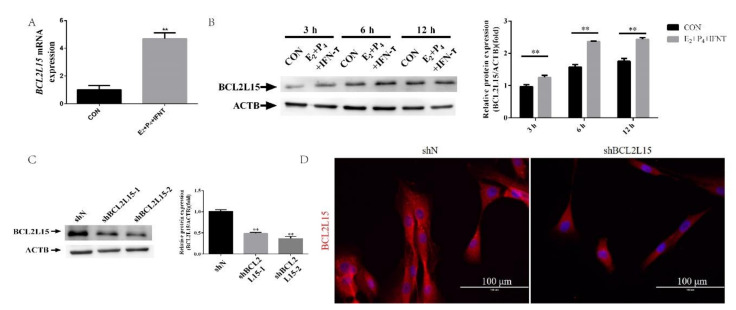
The expression of B-cell lymphoma 2 (Bcl-2)-like protein 15 (BCL2L15) in endometrial epithelial cells (EECs) under progesterone (P_4_), estradiol (E_2_), and interferon-tau (IFN-τ) treatments. (**A**) EECs were treated with or without hormones, followed by treatment with or without IFN-τ for 12 h, then collected for real-time quantitative PCR analysis. (**B**) EECs were treated with or without hormones, followed by treatment with or without IFN-τ for 3, 6, and 12 h, then collected for Western blotting. (**C**) The silencing efficiency of the BCL2L15 was measured in the EECs. The negative control short hairpin RNA (shRNA) and pCD513B-U6-BCL2L15-shRNA were transduced for 48 h. (**D**) Fluorescence microscope images of BCL2L15 expression in EECs. Scale bar = 100 μm. The data are presented as the means ± S.E.M. of three independent experiments. ** Significant difference (*p* < 0.01) compared with other groups.

We isolated mRNA and proteins from EECs to examine the BCL2L15 mRNA and protein levels in EECs under progesterone (P_4_), estradiol (E_2_), and IFN-τ treatments, which were adopted to mimic the in vivo intrauterine environment during the WOI [17,18,19]. Compared with the control (CON) group, the *BCL2L15* mRNA levels significantly increased at 12 h. Next, we assessed the BCL2L15 expression in protein extracts prepared from EECs at 3, 6, and 12 h under P_4_, E_2_, and IFN-τ treatments. We observed a similar expression pattern of the BCL2L15 protein. There was also a significant difference in the protein expression of BCL2L15 between the CON and E_2_+P_4_+IFN-τ groups at 3, 6, and 12 h (Figure 1B). To determine the impact of BCL2L15 in regulating the endometrial receptivity, two plasmids were constructed to inhibit the activity of BCL2L15, and a negative control plasmid was obtained from *E. coli*. These were named shBCL2L15-1, -2, and shN, respectively. As shown in Figure 1C, the shBCL2L15-2 group had the strongest effect on the down-regulation of BCL2L15. Further analysis showed similar phenomena. The BCL2L15 proteins were localized abundantly in the cytoplasm of EECs in shN, compared with shBCL2L15 (Figure 1D). 

### 2.2. Knockdown of BCL2L15 Impaired Endometrial Receptivity

**Figure 2:**
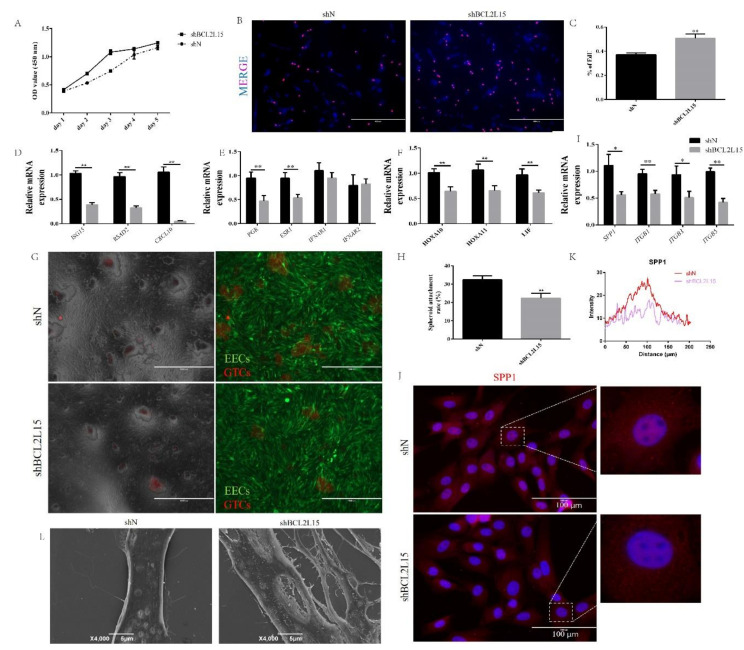
The effects of BCL2L15 on endometrial receptivity. The lentivirus specific for BCL2L15 and a negative lentivirus (shN) infected EECs for 48 h. (**A**) The cell proliferation was measured by Cell Counting Kit-8 (CCK-8) assay. (**B**,**C**) The DNA synthesis was measured by 5-ethynyl-2′-deoxyuridine (EdU) proliferation assay. (**D**–**I**) The EECs were treated with hormones followed by IFN-τ treatment for 12 h, then collected for real-time quantitative PCR. (**G**,**H**) Knockdown of BCL2L15 reduced the adhesion of goat trophoblast cells (GTCs) spheroids on EECs. Scale bar = 1000 μm. (**J**,**K**) Fluorescence microscope images of osteopontin (SPP1) expression in shN and shBCL2L15 EECs, which were treated with hormones followed by IFN-τ treatment for 6 h. Representative images of three independent experiments are shown. Scale bar = 100 μm. (**L**) Scanning electron microscopy (SEM) of EECs treated with hormones followed by IFN-τ treatment for 12 h. The data are presented as the means ± SEM of three independent experiments. * Significant difference (*p* < 0.05) compared with other groups; ** Significant difference (*p* < 0.01) compared with other groups.

Given that BCL2L15 expression was high in EECs under P_4_, E_2_, and IFN-τ treatments, we next wondered whether the BCL2L15 defects caused abnormal endometrial receptivity. Several lines of evidence suggested that the proliferation of EECs was inhibited, due to progesterone before the embryo attachment and implantation [20,21]. To evaluate the relationship between BCL2L15 and cell proliferation, we assessed the effects of a BCL2L15 knockdown on cell proliferation using a Cell Counting Kit-8 (CCK-8) and 5-ethynyl-2′-deoxyuridine (EdU) proliferation assay. As can be seen from Figure 2A, the proliferation rate of shBCL2L15 EECs was significantly higher than that of the control EECs. Consistent with the CCK-8 results, more EdU--positive cells were observed in BCL2L15-silenced EECs than negative EECs (Figure 2B,C).

To evaluate the receptivity, we examined the expression of ubiquitin cross-reactive protein (*ISG15*), radical S-adenosylcontaining domain 2 (*RSAD2*) and chemokine (C-X-C motif) ligand 10 (*CXCL10*), which were associated with promoting conceptus elongation [17]. We found that the shBCL2L15 group decreased the mRNA expression of *ISG15*, *RSAD2,* and *CXCL10* to a greater extent than found in the shN group (Figure 2D). During peri-implantation, hormones or IFN-τ bind to the cognate receptors for the regulation of physiological and pathological processes [22]. We next explored the mRNA level of receptors (*PGR*, *ESR1*, *IFNAR1,* and *IFNAR2*) between the shN and shBCL2L15 groups. Figure 2E shows that the reduction in *PGR* and *ESR1* was found compared with the shN group, but there were no significant differences in *IFNAR1* and *IFNAR2* between the shN and shBCL2L15 groups. We also found a knockdown of BCL2L15 reduced the expression of *HOXA10*, *HOXA11,* and *LIF*, which served as the key markers for endometrial receptivity [19,23] (Figure 2F). 

To further evaluate the receptivity, we investigated whether down-regulated BCL2L15 in EECs resulted in embryo implantation defects. The spheroid co-culture assay results demonstrated that the attachment of goat trophoblast cells (GTCs) spheroids (red) to shBCL2L15 EECs (green) was lower than that of shN EECs (Figure 2G and H). This result prompted us to measure the expression of the genes involved in cell adhesion. From Figure 2I, it can be seen that *osteopontin* (*SPP1*), *integrin β* (*ITGB*)*1*, *ITGB3*, and *ITGB5* were down-regulated by silencing BCL2L15. To further confirm the results, SPP1, a key cell adhesion molecule, was assessed using immunofluorescence. As expected, the shBCL2L15 groups showed a decreased fluorescence intensity of SPP1 compared with the shN groups (Figure 2J and K). In order to characterize the receptivity defects in shBCL2L15 EECs, we performed scanning electron microscopy (SEM) in shBCL2L15 and shN. SEM revealed that, in contrast to shN, the number of microvilli was substantially greater on the surface of shBCL2L15 EEC s (Figure 2L).

### 2.3. BCL2L15 Knockdown Activated STAT1 and STAT3 Pathways

**Figure 3:**
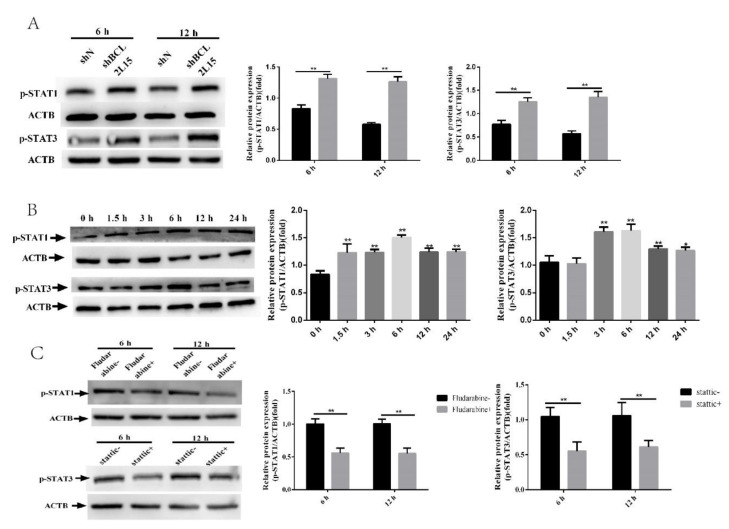
BCL2L15 affected the signal transducer and activator of transcription (STAT)1 and STAT3 pathways. (**A**) The lentivirus specific for BCL2L15 and a negative lentivirus (shN) infected EECs for 48 h. EECs were treated with or without hormones followed by treatment with or without IFN-τ for 6 h or 12 h, and then collected for Western blotting. (**B**) EECs were treated with or without hormones followed by treatment with or without IFN-τ for 0, 1.5, 3, 6, 12, and 24 h, then collected for Western blotting. (**C**) The shBCL2L15 EECs were pretreated with or without fludarabine and Stattic, respectively. Then, the EECs were treated with hormones, followed by treatment with IFN-τ for 6 h or 12 h, and then collected for Western blotting. The data are presented as the means ± SEM of three independent experiments. * Significant difference (*p* < 0.05) compared with other groups; ** Significant difference (*p* < 0.01) compared with other groups

To elucidate the molecular mechanism underlying the regulation of endometrial receptivity by BCL2L15, we detected the expression of p-STAT1 and p-STAT3 in shBCL2L15 EECs. The results presented in Figure 3A indicate that the shBCL2L15 EECs reported significantly more p-STAT1 and p-STAT3 than the shN EECs (Figure 3A). We monitored the expression of p-STAT1 and p-STAT3 at different time points. The Western blot results showed that p-STAT1increased after 1.5 h, and p-STAT3 was induced maximally at 6 h (Figure 3B).

### 2.4. Fludarabine Restored the Effect of Silencing BCL2L15 on *Endometrial Receptivity*

**Figure 4:**
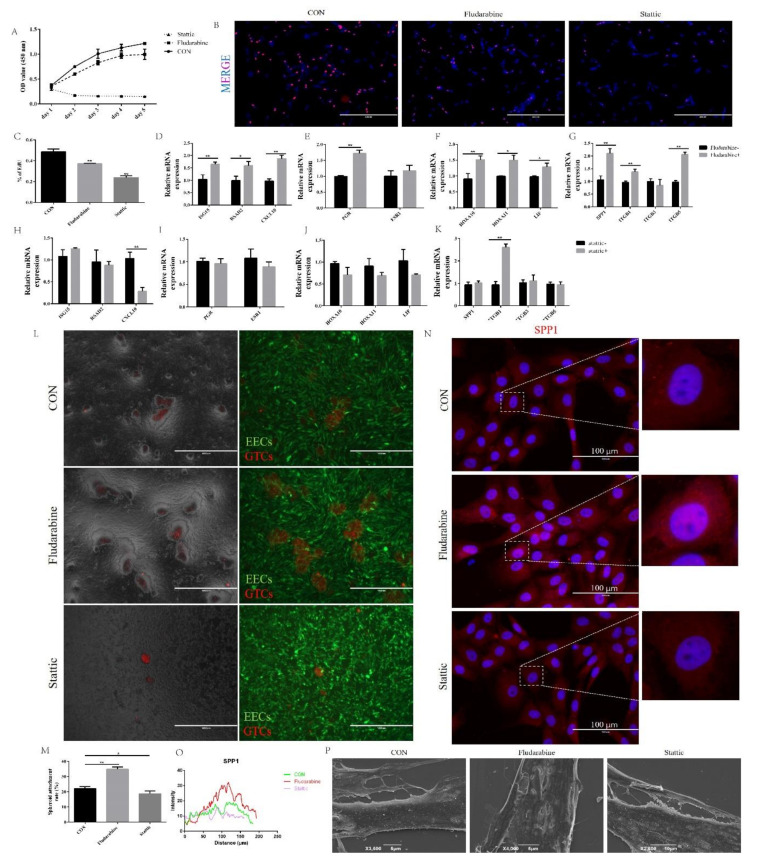
Inhibition of the STAT1 pathway suppressed BCL2L15 silencing-induced endometrial receptivity defects. (**A**) The cell proliferation was measured using a CCK-8 assay. (**B**,**C**) The DNA synthesis was measured using an EdU proliferation assay (**D**–**K**) The shBCL2L15 EECs were pretreated with or without fludarabine and Stattic. Then, the EECs were treated with hormones, followed by treatment with IFN-τ for 12 h, and then collected for real-time quantitative PCR. (**L**,**M**) Fludarabine treatment increased the adhesion of GTCs spheroids on EECs. Scale bar = 1000 μm. (**N**,**O**) Fluorescence microscope images of the SPP1 expression in the CON, fludarabine, and Stattic EECs, which were treated with hormones followed by IFN-τ treatment for 6 h. Representative images of three independent experiments are shown. Scale bar = 100 μm. (**P**) Scanning electron microscopy (SEM) of EECs, which were pretreated with or without fludarabine and Stattic. Then, EECs were treated with hormones followed by treatment with IFN-τ for 12 h. The data are presented as the means ± S.E.M. of three independent experiments. * Significant difference (*p* < 0.05) compared with other groups; ** Significant difference (*p* < 0.01) compared with other groups.

To scrutinize the role of the STAT1 and STAT3 pathway in the BCL2L15-regulation of endometrial receptivity, we treated EECs infected with shBCL2L15 with fludarabine or Stattic. As seen from Figure 3C, the expression of p-STAT1 and p-STAT3 were inhibited by fludarabine and static, respectively. The OD value is proportional to the number of cells. As shown in Figure 4A, the OD value was decreased by fludarabine in shBCL2L15 EECs. However, static-treatment clearly induced apoptosis (Figure 4A). EdU assays demonstrated that fludarabine or Stattic decreased the EdU-positive ratio (Figure 4B,C). The evaluation of the expression of promoting the conceptus elongation genes, *ISG15, CXCL10,* and *RSAD2*, indicated increased expression following treatment with fludarabine (Figure 4D).

Blocking the STAT1 pathway with fludarabine increased the *PGR* mRNA level, but no increase in *ESR1* was detected in the fludarabine group (Figure 4E). As shown in Figure 4F, higher transcription levels of *HOXA10*, *HOXA11*, and *LIF* were observed in the fludarabine treatment group compared with the control group. We also found that treatment with fludarabine promoted the expression of *SPP1*, *ITGB1*, and *ITGB5* mRNA, except in the *ITGB3* gene (Figure 4G). In addition, the treatment of shBCL2L15 EECs with fludarabine facilitated the adhesion of 34.9% of GTCs spheroids after spheroid placement on the shBCL2L15 EECs monolayer (Figure 4L and M). We also found that fludarabine promoted the distribution of SPP1 in shBCL2L15 EECs (Figure 4N and O). The SEM results showed that the microvilli on the surface of shBCL2L15 EECs flattened with fludarabine treatment. 

However, Stattic-treated shBCL2L15 EECs failed to reverse the BCL2L15 knockdown-induced endometrial receptivity defects. The qPCR results showed that no significant differences were found between the Stattic and CON groups (Figure 4H–K). For cell adhesion activity, there was no evidence that Stattic had an influence on the shBCL2L15 EECs adhesion (Figure 4L and M). Likewise, no significant difference in SPP1 expression between the two groups was evident (Figure 4N and O). We also found no significant reduction in Stattic-treated shBCL2L15 cell microvilli compared with the CON group. Overall, the STAT3 pathway did not affect the BCL2L15-mediated endometrial receptivity.

## 3. Materials and Methods

### 3.1. Cell Culture and Drug Treatment

Human telomerase reverse transcriptase (hTERT) was transfected into primary goat endometrial epithelial cells (EECs) and goat trophoblast cells (GTCs), to induce immortalization [24,25,26]. The EECs and GTCs were cultured in 6-well plates with DMEM/F-12 medium, supplemented with 10% fetal bovine serum (FBS, Biological Industries Israel Beit Haemek Ltd., Beit Haemek, Israel). 

The EECs were cultured in DMEM/F-12 supplemented with 0.1% bovine serum albumin (BSA) for 24 h, when EECs reached 70–80% confluence. Then, P_4_ (10^−7^ M, Sigma, St. Louis, MO, USA) and E_2_ (10^−9^ M, Sigma, St. Louis, MO, USA) were added to the medium. Following the 12 h treatment, the EECs were treated with 20 ng/mL IFN-τ (Sangon Biotech Co., Ltd., Shanghai, China) for 6 h or 12 h. In the presence of fludarabine (STAT1 inhibitor, ApexBio Technology, Houston, TX, USA) or Stattic (STAT3 inhibitor, ApexBio Technology, Houston, TX, USA), 10 μM fludarabine or 10 μM Stattic were added to the EECs, before the treatment of IFN-τ.

### 3.2. Cell Transfection

The recombinant lentivirus expressing short hairpin BCL2L15 (shBCL2L15) and negative (shN) were constructed and packaged according to a previous report [27]. The sequences of shBCL2L15 and shN are shown in Table 1.

### 3.3. Spheroid Co-Culture Assay

The GTCs were labeled with CellTracker CM-DiI (1 μM, Yeasen Biotech Co., Ltd., shanghai, China) and seeded at 2500 cells per well in non-adherent round bottom 96well plates, to encourage spheroid development. As Gabriella et al. described [28], spheroids (approximately 50 spheroids per well) were placed onto EECs that were treated with hormones followed by treatment with IFN-τ for 6 h. The EECs/GTCs spheroid co-cultures were shaken for 10 mins at 110 rpm after 1 h incubation. The number of GTCs remaining in wells were counted and the adhered spheroids expressed as a percentage of the seeded spheroids.

### 3.4. RNA Extraction and Real-Time Quantitative PCR

The total RNA was extracted using the TRIzol reagent (TaKaRa Bio, Inc., Dalian, China) and synthesized cDNA from the ABScript II RT Master Mix for qPCR (ABclonal Biotechnology, Wuhan, China). The primer sequences are listed in Table 2. Real-time quantitative PCR was performed using ABclonal 2X Universal SYBR Green Fast qPCR Mix (ABclonal Biotechnology, Wuhan, China) n Step One Real-Time PCR System (Applied Biosystems, Carlsbad, CA, USA). The 2^−△△Ct^ method was used to estimate the expression levels. The mRNA levels were normalized with the *GAPDH* gene.

### 3.5. Western Blot Analysis

The total proteins from the EECs were extracted using RIPA buffer (Beijing Solarbio Science & Technology Co., Ltd., Beijing, China). The BCA Protein Assay Kit (Nanjing Keygen Biotech Co., Ltd., Nanjing, China) was used to measure total protein concentration. 30 μg of protein were fractionated on 12% SDS-PAGE gel, transferred to PVDF membranes and blocked in 10% nonfat milk in Tris-buffered saline containing 0.5% Tween-100 (TBST). The PVDF membranes were incubated with anti-BCL2L15 (BIOSS bs-7582, 1:500), anti- phospho-STAT3 (Ty705) (CST 9145, diluted 1:1000), anti-phospho-STAT1 (Ser727) antibody (CST 9177, diluted 1:1000), and anti-ACTB antibody (Proteintech Group, Inc, diluted 1:2000) overnight at 4 °C. After incubating with an HRP-labeled secondary antibody, the protein expression was visualized using the Image-Pro plus 6.0 software (Media Cybernetics, Inc., Silver Spring, MD, United States).

### 3.6. Immunofluorescent Staining

The method was described in our previous publication [29]. The samples were blocked with 5% BSA in PBS for 1 h, followed by an incubation with primary antibodies (anti-BCL2L15 ((BIOSS bs-7582, 1:200); anti-SPP1 (Wanleibio Co., Ltd. WL02378, diluted 1:150)), and Alexa-labeled donkey anti-rabbit secondary antibodies. The nuclei were counterstained by 4’,6-diamidino-2-phenylindole (DAPI, Beyotime, Haimen, China) and observed using a fluorescence microscope (Nikon Inc, Melville, NY, USA).

### 3.7. Measurement of Cell Viability

We seeded the EECs in 96-well plates with 5 × 10^3^ cells/well, then added with 10 μL of CCK-8 (Beyotime, Haimen, Jiangsu, China) for 2 h. The OD value at 450 nm was measured using a Microplate Reader (Bio-Rad 680, Hercules, CA, USA).

### 3.8. EdU Proliferation Assay

We seeded the EECs in 24-well plates with 5 × 10^4^ cells/well, and cultured them as previously described [30]. After incubating with 10 μM EdU for 6 h, the EECs were fixed and washed with PBS containing 3% BSA. Then, the EECs were permeabilized and stained according to the manufacturer’s instructions. The samples were observed by fluorescence microscope (Nikon Inc, Melville, NY, USA). 

### 3.9. SEM Analysis

SEM analysis was performed following previous studies [31]. Briefly, the cell samples were fixed with glutaraldehyde for 48 h, then dehydrated in a graded ethanol series and critical point dried. The cell samples were coated with gold and observed with a JSM-6390LV.

### 3.10. Statistical Analysis

Unless otherwise specified, all data are expressed as the mean ± SD. One-way ANOVA, followed by Fisher’s least significant difference (LSD) or Student’s *t*-tests, was performed. Statistical differences were considered significant when the *P* value was less than 0.05.

## 4. Discussion

Endometrial receptivity is a prerequisite to render the uterus suitable for embryo adhesion and to establish pregnancy in ruminants [18,19]. Although the molecular mechanism of regulating endometrial receptivity remains unclear, it is well-documented that altered gene expression in EECs during early pregnancy guarantees the success of embryo implantation [32]. Previous studies reported that exosomal proteins derived from day 17 pregnant ewes up-regulated BCL2L15 protein expression [10]. The pronounced BCL2L15 expression change was observed between the pre-receptive endometrium and receptive endometrium in goats by Ribo-Zero RNA-Seq [16]. Here, we presented the results of the expression analyses of BCL2L15, and its role on its downstream target STAT1 on endometrial receptivity in vitro. Our findings showed that endometrial receptivity was regulated by BCL2L15. Given that BCL2L15 was upregulated under P_4_, E_2_, and IFN-τ treatments, the knockdown of BCL2L15 by specific shRNA significantly hampered the endometrial receptivity. By attenuating STAT1 signaling, fludarabine reversed the endometrial receptivity in shBCL2L15 EECs.

To elucidate the function of BCL2L15 on the endometrial receptivity, a lentiviral packaging system was employed to produce shBCL2L15 vectors. Our current study found that BCL2L15 silencing accelerated the cell proliferation. This finding is consistent with that of Ragusa, who found that BCL2L15 depletion promoted the clonogenic growth of intestinal organoids in vitro [15]. However, ceasing EEC proliferation is necessary for endometrial receptivity in response to P_4_. The qPCR results demonstrated that the low level of *PGR* in shBCL2L15 EECs was required to bind P_4_ during early pregnancy. We therefore that the speculate loss of BCL2L15 may impair this important step in P_4_ induced proliferation arrest. During peri-implantation, several genes, such as *ISG15*, *CXCL10*, and *RSAD2*, were assumed to promote the conceptus elongation and implantation [17]. 

Previous studies demonstrated that robust ISG15 expression in the endometrium may facilitate conceptus attachment or improve the resistance against infection [33]. Imakawa et al. reported that CXCL10 was detected in the uterine epithelium of pregnant goats, and that CXCL10 played an important role on embryo attachment by recruiting immune cells [34,35]. The function of RSAD2 on enhancing tolerance toward the conceptus, which may induce a physical relationship between the endometrium and conceptus, is widely known [36]. Here, we observed that the promoting conceptus elongation and implantation related genes were reduced when BCL2L15 was in knockdown. Therefore, it is possible that BCL2L15 is involved in promoting conceptus elongation and implantation during the maternal recognition of pregnancy. 

We wondered whether BCL2L15 affected the expression of the endometrial receptivity marker genes. Not surprisingly, reductions of *HOXA10*, *HOXA11*, and *LIF* expression were observed in shBCL2L15 EECs. The strength of cell adhesion directly affects the attachment of embryos [28]. With the wide application of spheroid co-culture assay, it is possible to measure cell adhesion in vitro. We demonstrated that the knockdown of BCL2L15 decreased the percentage of the attached GTCs spheroids to EECs and inhibited the expression of the adhesion molecules SPP1, ITGB1, ITGB3, and ITGB5.

Kaneko et al. reported that uterine epithelial cells undergo extensive morphological remodeling to a state that allows blastocyst attachment/invasion [37]. These changes are called “plasma membrane transformations”, and they include microvilli flattening [38]. Blastocysts preferentially implant to smooth cell surfaces rather than those covered by microvilli during early pregnancy [39]. We observed that shBCL2L15 EECs lost the ability of microvilli retraction. Together, these lines of evidence support a critical role of BCL2L15 in endometrial receptivity.

Previous work demonstrated that STAT1 and STAT3 are hormonally regulated in the endometrium [9,40], and involved in the physiological and pathological processes [2,3]. We wondered whether STAT1 and STAT3 served as downstream molecules in BCL2L15 mediated endometrial receptivity. The results of this study indicated that BCL2L15 negatively regulated the STAT1 and STAT3 pathways in EECs under P_4_, E_2_, and IFN-τ treatments. Previous studies showed that fludarabine treated HTR-8/SVneo cells only inhibited the STAT1 phosphorylation without changing the STAT1 protein status [41]. Du et al. reported that the protein level of STAT3, p-STAT3^Ser727^, and p-STAT3^Tyr705^ were inhibited by Stattic treatment in the MA-891 cells [42]. In deciphering the effects of the STAT1 and STAT3 pathways on endometrial receptivity during the peri-implantation period of pregnancy, we treated fludarabine and Stattic in shBCL2L15 EECs. Our results are in line with those of previous studies; p-STAT1 and p-STAT3 were blocked by fludarabine and Stattic, respectively, pre-treatment. 

Francisco’s group reported that pre-treatment with fludarabine reduced the human trophoblast cell line HTR-8/SVneo cell viability [43]. Consistent with the literature, our results found that shBCL2L15 EEC proliferation was inhibited by fludarabine treatment. However, Stattic induced significant apoptosis at 24 h in shBCL2L15; these results reflect those of Sruthi et al., who also found that Stattic induced bladder cancer cell apoptosis at 24 h by arresting the G2/M cell cycle [44]. Blocking the STAT1 pathway with fludarabine increased the expression of genes promoting conceptus elongation, endometrial receptivity markers, cell adhesion molecules, and PGR, except for ITGB3. 

An increase in the spheroid attachment rates and the translational level of SPP1 was observed by fludarabine treatment. In addition, fludarabine recovered the flattened microvilli on the surface of shBCL2L15 EECs. However, the STAT3 inhibitor failed to restore the abnormal endometrial receptivity caused by BCL2 deficiency. It can, thus, be suggested that the STAT1 pathway is a potential downstream signaling pathways of BCL2L15-regulated endometrial receptivity. It is unclear whether BCL2L15 regulates the STAT1 pathway involved in endometrial receptivity directly or indirectly. Further studies are required to verify more detailed molecular mechanisms.

## 5. Conclusions

In summary, this study demonstrated that BCL2L15 was activated by P_4_, E_2_, and IFN-τ treatments and that BCL2L15-STAT1 was involved in regulating the endometrial receptivity in goats. We also confirmed that BCL2L15 silencing promoted EEC proliferation, inhibited cell adhesion, and induced the loss of the ability for microvilli retraction. The requirement of STAT1 in the shBCL2L15 EECs suggested that the STAT1 pathway is required for endometrial receptivity. Understanding the role of BCL2L15-STAT1 in endometrial receptivity is critical to exploring new targets for the diagnosis and treatment of early pregnancy failure, and improving artificial reproduction success rates. Therefore, our data demonstrated a foundation for future studies aimed at developing new insights into the endometrial receptivity of ruminants.

## Figures and Tables

**Table 1:** Short hairpin interfering RNA (shRNA) inserts.

shRNA	Sequence (Loop in Bold Letters) (5′ to 3′)
shBCL2L15-1	GATCCGGTATCGAACACCAACTAAGCCTCGAGGCTTAGTTGGTGTTCGATACCTTTTTG AATTCAAAAAGCTGGGTGACAAATTCAATGGCTCGAGCCATTGAATTTGTCACCCAGCG
shBCL2L15-2	GATCCATTGGAAGCTTCTGCCAGAAACTCGAGTTTCTGGCAGAAGCTTCCAATTTTTTG AATTCAAAAAATTGGAAGCTTCTGCCAGAAACTCGAGTTTCTGGCAGAAGCTTCCAATG
shN	GATCCTTCTCCGAACGTGTCACGTTTCAAGAGAACGTGACACGTTCGGAGAATTTTTTG AATTCAAAAAATTCTCCGAACGTGTCACGTTCTCTTGAAACGTGACACGTTCGGAGAAG

**Table 2:** Primer pairs used for real time quantitative PCR.

Gene	Sequences (5′→3′)	References or GenBank Accession Number
*BCL2L15*	Forward: ctgtcctgccacgttaggat Reverse: tctctcagcaatgcctggt	XM_018045934.1
*ISG15*	Forward: ggtgaggaacgacaagggtc Reverse: cagaattggtccgcttgcac	XM_005690795.3
*CXCL10*	Forward: ggttttcttattttctgccttat Reverse: atccattactgatctcgatgc	NM_001285721.1
*RSAD2*	Forward: tgcttggtgcccgagtctaac Reverse: tccgcccatttctacagttca	XM_018055702.1
*PGR*	Forward: aagccagccagagcccacagt Reverse: tgcaatcgtttcttccagcacata	XM_018059880.1
*ESR1*	Forward: atcaactgggcaaagagggtg Reverse: aggttgggagcaaataggagc	XM_018053363.1
*IFNAR1*	Forward: aacctccttcctctgttgacg Reverse: ttgggaattgtactcttcgtg	XM_018046572.1
*IFNAR2*	Forward: cagcctcgtatttggtatttc Reverse: cagtccttgacgaccttcata	XM_018046609.1
*ITGB1*	Forward: tccctaagtcagcggtaggaa Reverse: tccggtaatttgctgtcctcc	NM_001285667.1
*ITGB3*	Forward: acggtgagcttcagcattga Reverse: acaccccacactcaaaggtc	XM_018047091.1
*ITGB5*	Forward: cccacgagaaggctacttgg Reverse: ttcaacaggcgtctcgatcc	XM_018047092.1
*SPP1*	Forward: tgagaattgcagtgatttgc Reverse: tgagatgggtcaggctttag	XM_005680968.3
*HOXA10*	Forward: cttccaaaggcgaaaacgca Reverse: gtctggtgcttggtgtaggg	XM_018047091.1
*HOXA11*	Forward: cagattcgggagctagagcg Reverse: cggtcagtgaggttgagcat	XM_018047092.1
*LIF*	Forward: cttccccaacaacctgga Reverse: gcgatgatgcgatacagc	XM_005691625.3
*GAPDH*	Forward: gatggtgaaggtcggagtgaac Reverse: gtcattgatggcgacgatgt	XM_005680968.3
